# When to start: not so fast

**DOI:** 10.7448/IAS.15.6.18071

**Published:** 2012-11-11

**Authors:** J Lundgren

**Affiliations:** Copenhagen University Hospital and University of Copenhagen, Copenhagen, Denmark

## Abstract

It remains controversial whether and, if so, the extent to which antiretroviral therapy (ART) results in net benefit if used by HIV-positive persons with a high CD4 count, particularly those with early HIV infection. This controversy is primarily reflecting lack of solid evidence from randomized controlled trials. Currently published trials have compared early ART with initiation of ART below currently globally accepted thresholds for initiation (i.e. CD4 count at 350 cells/µL) and, hence, are unable to inform this discussion. Analyses on large observational studies that have attempted to address this question have shown inconsistent results; therefore, those results are considered low-quality evidence, as per the GRADE criteria used by, for example, WHO when formulating guidelines. In resource-constrained regions, not even observational data are available to inform this question. The START study is underway to answer this question. Data remain blinded, but START may show net harm from early use of ART; such a result would severely undermine use of ART as prevention in early HIV infection. Prescription of any type of medicine is guided by the principle of “do no harm” – that is, “the doctor should not prescribe medications unless s/he knows that the treatment is unlikely to be harmful.” Hence, the balance of risk/benefit to individuals versus prevention benefit is important to accurately determine, and current guidelines of generally initiating ART once the patient develops HIV-related symptoms or the CD4 count drops to levels around 350 cells/µL should be adhered to until further evidence has emerged.

